# The Design and Experimental Development of Air Scanning Using a Sniffer Quadcopter

**DOI:** 10.3390/s19183849

**Published:** 2019-09-06

**Authors:** Endrowednes Kuantama, Radu Tarca, Simona Dzitac, Ioan Dzitac, Tiberiu Vesselenyi, Ioan Tarca

**Affiliations:** 1 Department of Electrical Engineering, Pelita Harapan University, Tangerang 15811, Indonesia; 2 Mechatronics Department, University of Oradea, 1 Universitatii St., Oradea 410087, Romania; 3 Energy Engineering Department, University of Oradea, 1 Universitatii St., Oradea 410087, Romania; 4 Department of Mathematics, Computer Science, Aurel Vlaicu University of Arad, St. Elena Dragoi, Arad 310330, Romania or; 5 R & D Center: “Cercetare Dezvoltare Agora”, Agora University of Oradea, St. Piata Tineretului, Oradea 410087, Romania; 6 Mechanical Engineering and Automotive Department, University of Oradea, 1 Universitatii St., Oradea 410087, Romania

**Keywords:** quadcopter, drone, pollutant, carbon, air monitoring, kernel

## Abstract

This study presents a detailed analysis of an air monitoring development system using quadcopters. The data collecting method is based on gas dispersion investigation to pinpoint the gas source location and determine the gas concentration level. Due to its flexibility and low cost, a quadcopter was integrated with air monitoring sensors to collect the required data. The analysis started with the sensor placement on the quadcopter and their correlation with the generated vortex. The reliability and response time of the sensor used determine the duration of the data collection process. The dynamic nature of the environment makes the technique of air monitoring of topmost concern. The pattern method has been adapted to the data collection process in which area scanning was marked using a point of interest or grid point. The experiments were done by manipulating a carbon monoxide (CO) source, with data readings being made in two ways: point source with eight sampling points arranged in a square pattern, and non-point source with 24 sampling points in a grid pattern. The quadcopter collected data while in a hover state with 10 s sampling times at each point. The analysis of variance method (ANOVA) was also used as the statistical algorithm to analyze the vector of gas dispersion. In order to tackle the uncertainty of wind, a bivariate Gaussian kernel analysis was used to get an estimation of the gas source area. The result showed that the grid pattern measurement was useful in obtaining more accurate data of the gas source location and the gas concentration. The vortex field generated by the propeller was used to speed up the accumulation of the gas particles to the sensor. The dynamic nature of the wind caused the gas flow vector to change constantly. Thus, more sampling points were preferred, to improve the accuracy of the gas source location prediction.

## 1. Introduction

Several types of air, water, and soil pollutants are impossible to avoid, being encountered in almost all countries. Some of them represent real threats, rising risks to human health and environment degradation, such as (for the case of big cities) air pollution generated by stationary sources (e.g., factories, power plants), by mobile sources (e.g., cars, buses), and also natural sources (e.g., windblown dust, wildfires). Pollutants can be classified into two types: pollutants having known sources, and pollutants having unknown sources. Each of them have specific approaches related to measurement methods and source identification. Most developed countries have developed law regulations to organize regular air quality supervision for primary urban pollutants [1,2,3]. The regulations could be put into practice only by monitoring regularly and accurately the concentration of the pollutant. Usually, stationary monitoring stations with network systems located in critical areas for data collection and processing are used [4,5,6]. This type of monitoring is limited by the sampling location and data accessibility [7]. With the advance in technology, this old paradigm gets surpassed by the utilization of sensor technology which is superior in respect to the low-cost material, simplicity and affordability, and also the portability of the air pollution monitoring systems [8,9]. The technology above can be integrated into an air scanning system by using an unmanned aerial vehicle (UAV) characterized by good maneuverability, controllable altitude, and location. [10,11]. Although the recent developments in UAV technology are promising, there are studies that show that in particular areas of applications (for example environmental restoration monitoring) there are still many issues to be addressed [12]. A comprehensive review of using autonomous vehicles in environmental monitoring for pollution source localization is presented in [13]. A design and manufacturing protocol for the low cost and low weight quadcopter platform prototype for the purpose of environmental monitoring and research in order to assess ecological devastation of the natural environment is presented in [14].

Different recently published studies show the interest of researchers in the applications of quadcopters used in different fields of environmental monitoring like sampling of different substances, monitoring soil contamination, and response to natural disasters. Lally, H.T. et. al. [15] addresses the development of water sampling by using drones specially equipped with water-sampling devices. The issues of biological and physico-chemical sampling are described as well as some solutions for these issues. The authors show that it is envisaged that drone-assisted water sampling will act as a pivotal supporting tool if the cost benefit analysis of the application gives positive results. In [16] the author’s goal was to introduce and test a method able to predict copper accumulation points, using aerial photos taken by drones and micro-rill network modeling. In this case the drone collected photogrammetric data, which was compared with the results obtained by computer modeling. According to the results of the study, the authors were able to predict zones of copper accumulation at a plot scale. Other important applications of drones are presented in [17] where is stated that UAV’s can have a crucial role in the case of natural disaster response and humanitarian relief aid. The key areas of intervention in this case are: aerial monitoring of post-natural disaster damage, natural disaster logistics and cargo delivery, and post-natural disaster aerial assessment. An application which is close to our paper’s subject is presented in [18] where gas concentrations resulting from an underground coal fire (carbon dioxide emissions) were measured using aerial monitoring with drones. The authors state that it is estimated that these fires generate as much as 3% of the world’s annual carbon dioxide emissions and that drone collected gas concentration data provides a safe alternative for evaluating the rank of burning coal deposits.

A portable air scanning system was developed using a quadcopter equipped with an air scanning sensor to perform air quality measurement, thus called a ‘sniffer’ quadcopter. The development began with analysis of the correlation between propeller’s air trajectories and the sensors placement, determination of an appropriate flying pattern to optimize the air measurement, and investigation of ways to minimize wind effects in the measurement process. A computational method was used to ascertain the sensor’s placement on the quadcopter, and the result was proven in the field test. The low-cost and portable gas sensors MQ-2 and MQ-135 were used to measure carbon concentration in the air. To develop this system, the sensor placement in the quadcopter is critical. Using computational fluid dynamic (CFD) simulation, the vortex field generated by the propeller was analyzed to determine the best place for sensor mounting. With an appropriate mounting place, the response time and the accuracy of data collected by the sensor can be increased [19]. Even with low-cost instruments, the data accuracy and detection range can be as good as conventional monitoring [20]. Two types of flight patterns for air measurement were used to detect the direction of gas dispersion and discover the gas source location and gas concentration level. The present system was designed to perform flight pattern measurement methods which consist of point source measurement and non-point source measurement. A path planning with eight sampling points around the gas source was used to obtain the gas concentration at a known point source, for example, an industrial emission or chimney [21]. The sample points formed a square with the gas source in the middle. Thus, more accurate data can be obtained despite the dynamic environmental change. On the other hand, for a non-point or unknown sources such as forest fires or pipe gas leaks, a grid pattern with 24 sampling points was used to detect the gas source location based on the gas dispersion measurement and analysis. To optimize the measurement results, wind effect was considered. Gas source location and gas concentration level can only be estimated with statistical methods. In the case of fires, an extensive study had been made to detect sources with UAVs equipped with thermal detection capabilities [22]. Gas concentration detection (for example CO concentration) can give additional information besides the thermal data. The method described in this paper is much more cost effective and can be used by smaller communities (at the level of small cities and towns) to check for fire sources that can be a threat for the population or economy. The results from both methods were evaluated using analysis of variance (ANOVA) to obtain the gas concentration at the source, and using a Gaussian dispersion model to analyze the gas dispersion. In the Gaussian dispersion model, parameters such as wind speed and direction, source term, etc., were obtained by monitoring data to acquire positioned trajectories with bivariate input environmental data [23,24]. In a small scale of gas measurement (<100 m), a sparse Gaussian kernel method was used as a statistical evaluation on a two-dimensional spatial model of a grid pattern to deal with the specific properties of gas dispersion, including the turbulent features of the wind [25,26]. Spatial integration is made by convolving sensor readings and modeling the information data of the point measurements with a Gaussian kernel method. The grid pattern size and data retrieval time depend on the sensors’ sensitivity. This research is limited with the usage of low-cost sensors and a narrow pattern, but these don’t affect our goal which is to prove that the Gaussian kernel method is suitable to analyze gas dispersion vectors and detect gas source locations.

The sniffer quadcopter was designed to work automatically according to the command input in the pre-flight setting, one of which is a GPS coordinate. In automatic mode, the quadcopter will fly to the target point to perform the measurement with the pre-programmed flight pattern. In this research, the measurement target is carbon monoxide (CO) because it was easily found and/or made and was measured with low-cost and portable gas sensors MQ-2 and MQ-135. The tests were performed on an aerial zone with a maximum of 24 sampling points, each measuring 1 m^2^. On each point, the sampling time was 10 s and the data was collected whilst maintaining the quadcopter in hover mode. The aerial zone was intentionally small so the research could be focused on the algorithm for gas source determination and gas concentration level, besides optimizing the sensor placement on quadcopter. The main purpose of our paper is to study the possibility of using low-cost aerial monitoring system and pollution source detection, which can be available to smaller communities (towns, NGOs, environmental protection associations) in order to detect and prevent threats to the environment.

## 2. Sniffer Quadcopter Design and Analysis

Prior to a quadcopter design, it is imperative to know the application of the drone itself, thus one can estimate the required lifting force in accordance to the total weight of the quadcopter. The physical factors of quadcopter design related to lifting force are propeller diameter, propeller’s angle of attack, quadcopter size, and rotor angular velocity. Greater lifting force and high-speed flight are not synonymous with larger propellers or high-speed rotors because they can cancel out the advantages of using a quadcopter. For example, an oversized propeller produces substantial air flows which can cause turbulence and flight instability.

To optimize the sniffer quadcopter design, a detailed analysis of the vortex field generated by the propeller’s angular velocity and its effect on sensor placement on the quadcopter was performed. This research used 13-inch propellers, a 380 rpm/v rotor, and 18.5-volt lithium polymer (LiPo) batteries. With this specification, it can be deduced that the maximum no-load rotor speed is 7030 rpm. To get a more accurate calculation of the lifting force, the computational fluid dynamic (CFD) module of the SolidWorks software was used. Table 1 shows detailed specifications of the quadcopter. The frame size was 460 mm, the quadcopter weight was 1800 g, and the sensor weight was 100 g. Quadcopter flight time depends on the capacity of the LiPo batteries used and the maximum carriable load depends on the total lifting force. The magnitude of the force analyzed with the CFD method and the correlation between thrust, frame size, and level of stability will be explained in detail in the propeller vortex field section.

**Figure 1:**
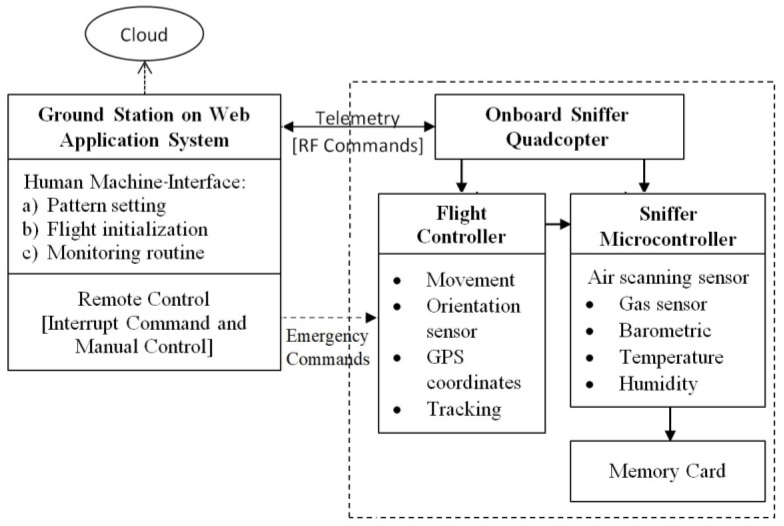
Sniffer quadcopter system architecture.

Due to short flight duration (<40 min), several settings must be initialized to permit the quadcopter to perform optimally, such as the target point, in the form of GPS coordinate, and quadcopter flight behavior, in the form of altitude, speed, and measurement pattern. The settings can also be made on the ground station system through the designed mission planner system which can communicate with the sniffer quadcopter using 433 MHz telemetry [27,28]. The architecture of the aerial platform system is presented in Figure 1. The ground station using an open source web application platform was also used to monitor all measurement processes. With the autonomous flight pattern, the quadcopter will run the sequential process according to the pre-flight command list. For intentional interruption of the process, an emergency system was designed so the user’s command can be delivered via the ground station control which will force the quadcopter either to make a landing or to fly back to the home coordinates. On board the sniffer quadcopter system one can find two controllers: the flight controller and sniffer microcontroller. The flight controller serves to maintain the stability of the maneuvers, together with an orientation sensor and a tracking pattern based on the input coordinate [29]. The sniffer system serves to perform air scanning and save the data on a memory card. Both controllers are always communicating with each other to determine when to carry out the data retrieval process.

### 2.1. Vortex Field Analysis

**Figure 2:**
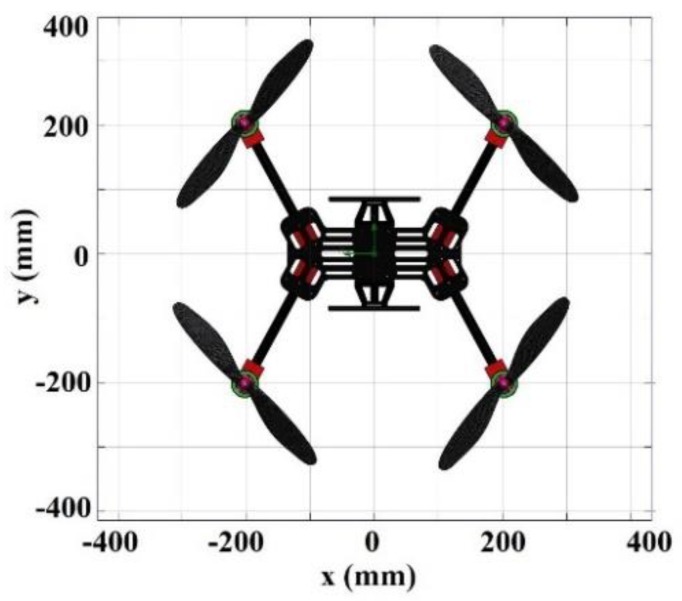
The sniffer quadcopter design.

Data retrieval was performed while keeping the quadcopter in the hover state at each analyzed point. It was noticed that data were profoundly affected by the air trajectories of the propeller’s vortex field and also by the wind. The vortex method was applied [30,31] to analyze the aerodynamic behavior of the aircraft. The authors’ previous studies [32,33] explain the quadcopter’s propeller design and flight stability analysis. As mentioned previously, the maximum no-load rotor speed was 7030 rpm. When connected with a 13-inch propeller, the maximum rotor speed was 4080 rpm. To obtain the total thrust, a CFD analysis was performed using the SolidWorks software. The computationally analyzed parameter was the propeller rotation with a set up rotation area, with 0.1% turbulence intensity with 0.002 m turbulence length, at a thermodynamic pressure of 101,325 Pa and a temperature of 293.2 K. Each propeller had a rotation region, in which a diagonal pair of propellers rotated clockwise (CW) and the other diagonal pair rotated counterclockwise (CCW) with a velocity of 0–4080 rpm. The simulation generated the values of the vertical force and the total air velocity in all propeller rotation areas. With a velocity of 4080 rpm, each rotor can produce a total thrust of 24 N. Besides that, the vortex field was also modeled using the same software with the same parameters. Figure 2 below shows the design of the sniffer quadcopter.

**Figure 3:**
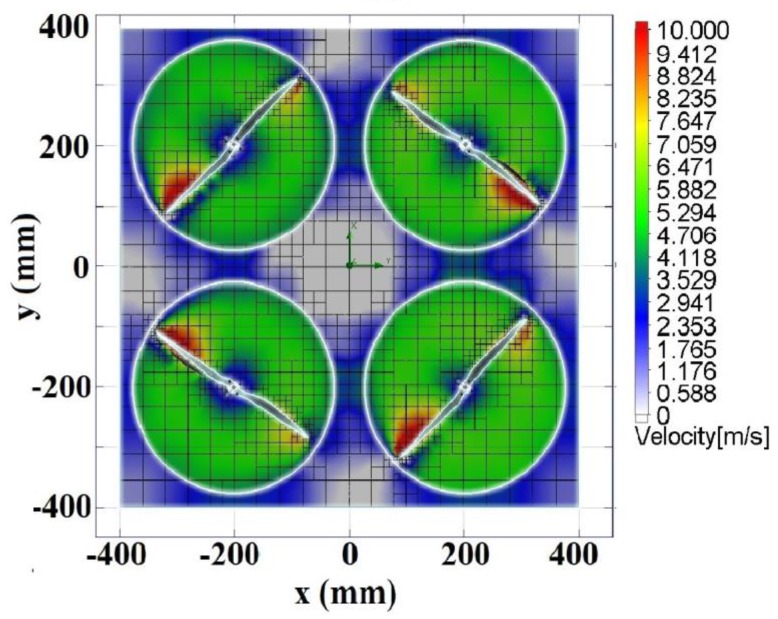
Air velocity dispersion.

In this study, the generated vortex field and its correlation with sensor position and the effect of the environmental change have been analyzed. The vortex field was modeled using computational fluid dynamics (CFD). The results showed that the air trajectories generated by each propeller rotation with maximum velocity didn’t affect each other because the air velocities produced were the same. Figure 3 presents the analysis of the vortex field using CFD. The propellers having a maximum angular velocity $\left(\Omega_{\max}\right)$ of 4080 rpm generate an air velocity of 6 ms^−1^ $\left(\upsilon_{\mathrm{air}}\right)\;$oriented downwards (along the *z*-axis); the air velocity measured between propellers (−30 ≤ x ≤ 30) mm and (−30 ≤ y ≤ 30) mm was 0.5 ms^−1^. For this study, we used a sniffer quadcopter with a total load of 1900 g keeping it in hover state at 80% of the maximum speed, which corresponds to 3264 rpm. Consequently, the angular velocity generated an air velocity of 4.8 ms^−1^ on the propeller, but the vortex fields generated by the propellers did not affect each other.

This means that the vortices generated during the hover state with no wind effects didn’t lead to turbulence on the quadcopter frame, which proved that the selected propeller diameter, quadcopter size, and rotor speed were appropriate. In the presence of wind effects, the quadcopter control system will work to maintain stability.

### 2.2. Correlation Between Vortex Field and Sensor Position

The transport of the monitored gas towards the gas sensor is a critical process to obtain accurate data which can be easily affected by the disturbances generated by the propeller’s vortex. Sensor position, gas distribution, and wind resistance are factors which influence the measurement results [34,35]. The downside of using a low-cost gas sensor is the low response time. To determine the sensors’ placement, one must consider the sensors’ response time whilst still maintaining the quadcopter stability. The use of an extended pole to place the sensors outside of propeller vortex field is not advisable because it can affect quadcopter stability. Even though it might not matter much with a small-scale sensor, an extended pole attached with a heavier sensor will surely be effect by the wind. Thus, the design and computational analysis for the gas scanning sensors’ placement on the quadcopter’s frame were only tested for two points, i.e., point A and point B.

**Figure 4:**
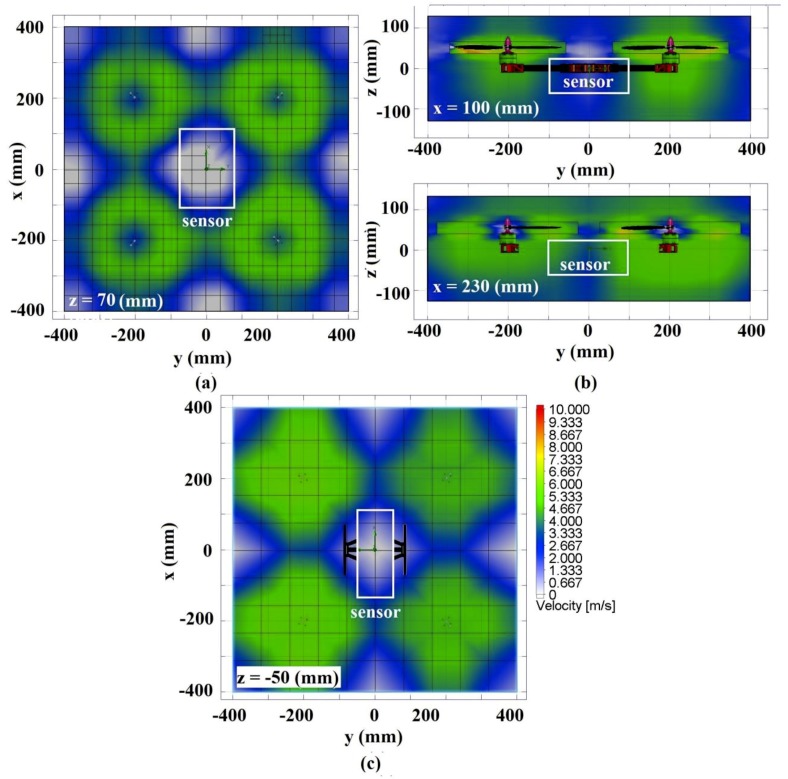
Air velocity on quadcopter in various perspectives: (**a**) Point A—top view; (**b**) Point B—front view; (**c**) Point A—bottom view.

Point A corresponds to the placement of the sensors at the bottom of the main frame; thus, the gas flow is not influenced by the propeller’s vortex. Point B corresponds to the situation in which the sensors are mounted on the front side of the frame so that the propeller’s vortex blows the gas directly on the sensors. Both positions were analyzed using CFD prior to the field tests. The results of the simulation process for the case of the propeller’s maximum angular velocity (4080 rpm) are presented in Figure 4. It can be noticed that the maximum air velocity occurs in each propeller rotation area while the minimum corresponds to the center of the frame. The placement of the air scanning sensor relies heavily on its type and response time. The following coordinates describe the point A’s position: (−50≤y≤50) mm; (−130≤x≤130) mm, and (z> −50) mm, where air speed is $\left(\upsilon_{\mathrm{air}}\approx 0{\;\mathrm{ms}}^{-1}\right)$ as seen in Figure 4c.

It is evident that at point A, each sensor is surrounded by the propeller’s vortices, with air trajectories going downward to produce thrust. For the analyzed gas to reach the sensor while the quadcopter is in the hover state, it is essential that the gas velocity be higher than the air velocity generated by the propeller. The vector of gas velocity towards the sensor, and the magnitude of total resultant velocity are very crucial to determine whether the gas can reach the sensor or not. Another way is to place the sensor on the top of the frame (z>70) mm or higher than the propeller as seen in Figure 4a. Point B corresponds to the placement of the sensor on the front of the frame (x>100) mm and takes advantage of the air trajectories generated by the propeller, as shown in Figure 4b. In this position, the gas around propeller is suctioned out by the propeller and passed through the sensor before going downward. This placement facilitates the gas to reach the sensor and is suitable for sensors with low response time.

**Figure 5:**
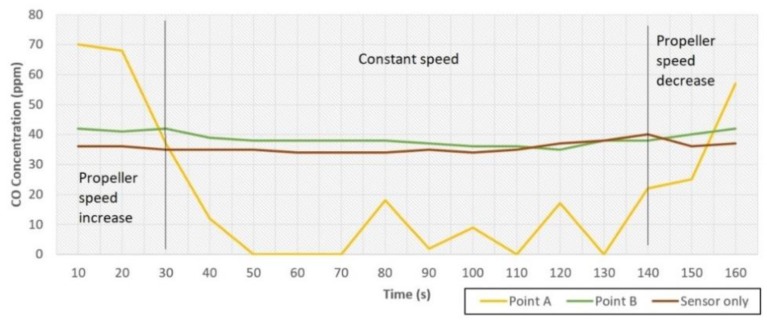
Comparison of sensor positions.

Figure 5 shows the comparison between three sensor placements in a field test with durations of 160 s and a stable gas source position. The aim of this test was not to compare the reading of gas concentration, but rather to see the sensors’ response time in different mounting positions. Analysis of point A showed that the gas concentration decreases as the speed of the propeller increases, and the gas concentration increases as the speed of the propeller decreases. This result was linear with the CFD analysis results. This means the sensors were obstructed by the propeller’s air trajectories and thus unable to properly measure the gas concentration. On the other hand, analysis of point B showed that the sensors’ reading was a bit affected by propeller speed change, but the sensor could work well when the propeller speed was stable. This was due to the propeller’s air flow ‘directing’ the gas towards the sensor. Lastly, the analysis was conducted on sensors only. The result showed the most stable measurement because it didn’t get affected by the propeller’s air trajectory. In conclusion, taking advantage of the propeller’s air trajectory to ‘direct’ the gas toward the sensors resulted in valid data reading, on the condition that the measurement was done while the quadcopter was in a hover state to ensure stable propeller speed in every measurement.

## 3. Grid Pattern Analysis

**Figure 6:**
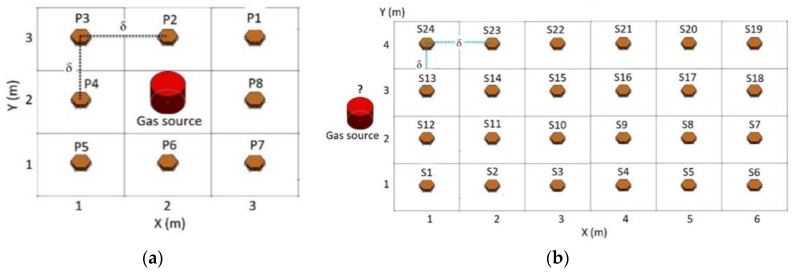
Pattern model with (**a**) point source, (**b**) non-point source.

The grid pattern and wind algorithm were integrated into the gas measurement process which was dynamically distributed [36]. Measurements can be performed for the case of either point source or non-point source. The point-source measurement is used for gas emissions with known locations, for example, chimney exhaust gases in industrial districts. However, as the gas dispersion depends on the wind direction an eight-point (P1–P8) square pattern is used to cover all wind blowing directions, as illustrated in Figure 6a. On the other hand, non-point source measurement is used to locate the gas source based on the particle density and wind direction. Data acquisition for this method allows the user to observe the gas dispersion gradient toward the closest point to the source (which has the highest particle concentration), exemplified in Figure 6b. In the field test, the grid pattern with 24 sample points (S1–S24) placed in a 4 × 6 matrix was able to be extrapolated using the Gaussian kernel method. The size of the cell depends on the sensitivity of the gas sensor.

The quadcopter’s flying sequence and sample points took place according to the grid numbers shown in Figure 6. The methodology consisted of collecting sample data of gas concentration $\left(G_{s}^{\left(i\right)}\right)$ on each cell k, at the location $x^{\left(i\right)}$ where the symbol *i* represents the number of the measurement sample.

As we mentioned above, sometimes position error occurred while collecting data. Thus, position adjustments (Δ*G*) were needed to ascertain that the measurements were made in the center of each grid cell (*G_c_*). The post-processing data errors relative to positioning errors on each cell can be minimized by data recovering process using Equation (1). The symbol *k* represents the number of the grid cell.

(1) $$\Delta G_{i}^{\left(k\right)}=\left|G_{c}^{\left(k\right)}-G_{s}^{\left(i\right)}\right|,$$

where, for the k cell, in which a number of $m^{\left(k\right)}$ measurements were made, $G_{c}^{\left(k\right)}$ value can be estimated with

(2) $$G_{c}^{\left(k\right)}=\frac{\sum_{i=0}^{m^{\left(k\right)}}G_{s}^{\left(k\right)\left(i\right)}}{m^{\left(k\right)}}$$

The differentiation of gas concentration between the center of the grid cell and the value acquired by the sensor was used to locate the gas source. If the relationship $\left({\Delta G}^{\left(k\right)}>\Delta G^{\left(k-1\right)}\right)$ is true, it means that the flight pattern moves toward the gas source, and conversely $\left({\Delta G}^{\left(k\right)}<\Delta G^{\left(k-1\right)}\right)$ means that the flight pattern moves away from the gas source. This post-processing of gas concentration variation in each cell helps in understanding the correlation between flight position and gas dispersion. In order to analyze the gas dispersion behavior, both for point source and non-point source models, an adaptive threshold with binary sample was used, as seen in Equation (3) [37].

(3) $${\bar{P}}^{\left(k\right)}={\bar{S}}^{\left(k\right)}=\{\begin{array}{l} 1\\ 0 \end{array}\begin{array}{l} \to (\Delta G_{t}^{\left(k\right)}>\Delta G_{t}^{\left(k-1\right)})\\ \to \left(\Delta G_{t}^{\left(k\right)}\leq \Delta G_{t}^{\left(k-1\right)}\right) \end{array}$$

For both methods, sample acquisition was done with the same iteration (t) The first value of gas concentration acquired has been used as the reference for measurements. The $\left({\bar{P}}^{\left(k\right)\;}={\bar{S}}^{\left(k\right)}=1\right)$ indicates an increase in gas concentration, whereas $\left({\bar{P}}^{\left(k\right)}={\bar{S}}^{\left(k\right)}=0\right)$ indicates a decrease.

### 3.1. Gas Dispersion

In order to analyze the gas dispersion measured by the quadcopter, a statistic method has been used to generate a two-dimensional gas distribution map, using the DM + V kernel algorithm presented in [38,39]. This algorithm treats the gas distribution model as a density estimation problem which can be solved using convolution with a two-dimensional Gaussian kernel. The kernel’s shape regulates the amount of extrapolation. When the wind is not blowing, the kernel’s shape is a circle, thereby: $\sigma_{x}=\sigma_{y}=\sigma_{0}$.

The vector of gas dispersion with spatial extrapolation was analyzed using the Gaussian weighting function (Ɲ) which represented the importance of the gas reading value obtained for each cell. The first step of the algorithm is the weight calculus $f_{i}{}^{\left(k\right)}\left(\sigma_{0}\right)$, which, intuitively represents the information content of a single measurement, *i,* of the sensor inside a net’s cell. The weight is calculated by the mean of a Gaussian kernel (N) evaluation applied to the distance between measurement location $x^{\left(i\right)}$ and the center point $x^{\left(k\right)}$ of the *k* cell.

(4) $$w_{i}{}^{\left(k\right)}\left(\sigma_{0}\right)=N\left(\left|x^{\left(k\right)}-x^{\left(i\right)}\right|,\sigma_{0}\right)$$

Starting from equation (4), the following values are integrated and placed in a temporary grid map: weights $W^{\left(k\right)}\left(\sigma_{0}\right)$, weighted sensor readings $G^{\left(k\right)}\left(\sigma_{0}\right)$, and weighted variance contributions $V^{\left(k\right)}\left(\sigma_{0}\right)$, as follows:

(5) $$W^{\left(k\right)}\left(\sigma_{0}\right)=\overset{n}{\underset{i=1}{\sum }}w_{i}{}^{\left(k\right)}\left(\sigma_{0}\right)$$

(6) $$G^{\left(k\right)}\left(\sigma_{0}\right)=\overset{n}{\underset{i=1}{\sum }}w_{i}{}^{\left(k\right)}\left(\sigma_{0}\right)G_{s}^{\left(i\right)}$$

(7) $$V^{\left(k\right)}\left(\sigma_{0}\right)=\overset{n}{\underset{i=1}{\sum }}w_{i}{}^{\left(k\right)}\left(\sigma_{0}\right)\tau^{\left(i\right)}$$

where $\tau^{\left(i\right)}={\left(\Delta G_{i}^{\left(k\right)}\right)}^{2}$ is the variance contribution of reading i.

From integrated weight map $W^{\left(k\right)}\left(\sigma_{0}\right)$ a confidence map ${\bar{x}}^{\left(k\right)}\left(\sigma_{0}\right)$ can be obtained showing the degree of trust with which for one cell the readings is considered to be in the vicinity of the respective grid cell’s center and is expressed as shown in the Equation (8).

(8) $$w_{i}{}^{\left(k\right)}\left(\sigma_{0}\right)=N\left(\left|x^{\left(k\right)}-x^{\left(i\right)}\right|,\sigma_{0}\right)$$

In normal dispersion, the confidence value is within the interval (0–1) which can be affected by the trajectory of the quadcopter, the size of the grid cell, the width of the kernel $\left(\sigma_{0}\right)$, and the scaling parameter $\left(\sigma_{r}\right)$.

Normalizing the integrated weighted sensor readings $G^{\left(k\right)}\left(\sigma_{0}\right)$ with the integrated weights $W^{\left(k\right)}\left(\sigma_{0}\right)$, then applying the confidence value and adding with the best guess for the cells with a low confidence (i.e., for cells for which we do not have sufficient information from nearby readings, indicated by a low value of ${\bar{x}}^{\left(k\right)}$) results in the map estimation of the mean distribution $g^{\left(k\right)}\left(\sigma_{0}\right)$:

(9) $$g^{\left(k\right)}\left(\sigma_{0}\right)={\bar{x}}^{\left(k\right)}\frac{G^{\left(k\right)}\left(\sigma_{0}\right)}{W^{\left(k\right)}\left(\sigma_{0}\right)}+\left\{1-{\bar{x}}^{\left(k\right)}\right\}G_{0}$$

As the best guess of the mean concentration $G_{0}$ we use the average over all sensor readings.

In the same way, the corresponding variance map $v^{\left(k\right)}\left(\sigma_{0}\right)$ results from normalizing the weighted variance contributions $V^{\left(k\right)}\left(\sigma_{0}\right)$ with the integrated weights $W^{\left(k\right)}\left(\sigma_{0}\right)$ then multiplying with the confidence value and adding with a best estimate for the cells with a low confidence:

(10) $$v^{\left(k\right)}\left(\sigma_{0}\right)={\bar{x}}^{\left(k\right)}\frac{V^{\left(k\right)}\left(\sigma_{0}\right)}{W^{\left(k\right)}\left(\sigma_{0}\right)}+\left\{1-{\bar{x}}^{\left(k\right)}\right\}v_{tot}$$

The estimate $v_{tot}$ of the distribution variance in regions far from measurement points is computed as the average over all variance contributions.

The mean value of gas concentration from each cell was used to make a predictive model in ANOVA. The spatial structure of the dispersion variance provided information on the gas dispersion vector and on the highest gas concentration which surely is located near the source.

### 3.2. Correlation Between Wind and Gas Dispersion

**Figure 7:**
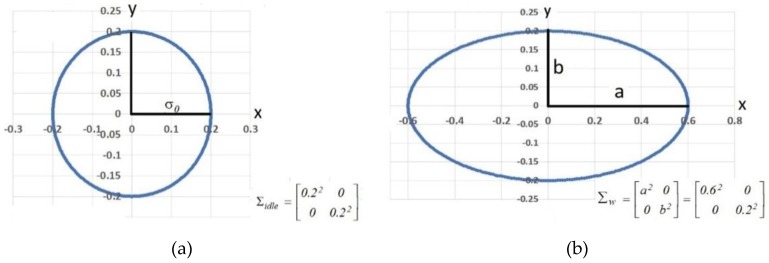
Wind direction model: (**a**) Idle state and (**b**) model considering wind velocity and direction.

The gas dispersion is in linear correlation with the wind dynamic movement vector. Knowledge about the wind vector is helpful in locating the gas source. The extrapolation of gas measurement using bivariate Gaussian kernel provides information about the wind vector [10,40]. Two possible models were considered, i.e., an idle state in which wind velocity is zero, and a windy state, correlated with wind direction. An example of a detailed model is presented in Figure 7.

The idle state with zero wind velocity is obtained from a normal dispersion having symmetrical kernel width $\left(\sigma_{0}\right)$ along the *x*-, *y*-, and *z*-axis on grid cell; a diagonal matrix with variance data (Σ) represents this state as seen in Equation (11).

(11) $$\Sigma_{idle}=\left[\begin{array}{cc} \sigma_{0}^{2} & 0\\ 0 & \sigma_{0}^{2} \end{array}\right]$$

Wind velocity creates gas dispersion in the form of an ellipse with linear dependency. The wind vector changes the ellipse position according to the amount of change in the rotation matrix R(α). Rotation matrix is an orthogonal matrix in which $R{\left(\alpha \right)}^{-1}=R{\left(\alpha \right)}^{T}$ rotate bivariate Gaussian kernels around the *x*- and *y*-axis. Angle alteration (α) in the horizontal position (*x*- and *y*-axis) determines a two-dimensional wind vector which can be calculated using Equation (12). Data was enough to determine the pollutant source using the grid cell.

(12) $$\sum_{R}=\left[\begin{array}{cc} \sigma_{x}^{2} & \alpha \sigma_{x}\sigma_{y}\\ \alpha \sigma_{x}\sigma_{y} & \sigma_{y}^{2} \end{array}\right]=R\left(\alpha \right)\Sigma_{w}R{\left(\alpha \right)}^{T}$$

(13) $$\;R\left(\alpha \right)=\left[\begin{array}{cc} \cos\alpha & \left(-\sin\alpha \right)\\ \sin\alpha & \cos\alpha \end{array}\right]\;$$

(14) ∑w=[a200b2]=[(σ0+γ|ν→|)200σ01+γ|ν→|σ02]

The gas dispersion along with the wind velocity and vector $\gamma \left(\vec{\nu }\right)$ were obtained from the wind sensor’s measurements located on the gas dispersion contour; *x*-axis values were proportional to the wind velocity, while *y*-axis values decreased with the wind velocity. The variable γ is the stretching parameter which depends on many environmental variables. The bivariate Gaussian kernel was rotated according to the wind vector.

## 4. Environmental Monitoring

**Figure 8:**
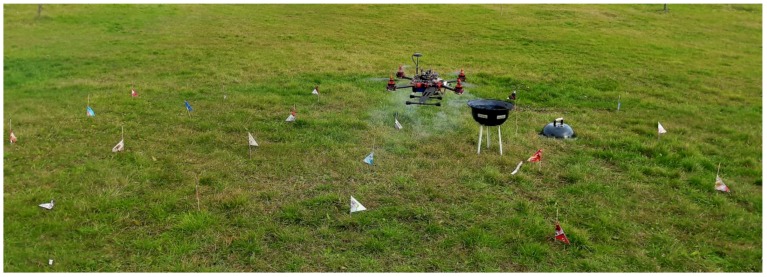
Sniffer quadcopter field test.

The experiment for measuring carbon concentration on each sample point was done in an open field for both the point source and the non-point source case. The pollutant source consisted of burning coals placed in a burner with a height of 0.5 m. The 24 grid cells covered an area of 6 m (width) × 4 m (length). Each cell was 1 m^2^ in size, and the distance between measurement points was also 1 m. This distance depends on the sensor sensitivity; the more sensitive the sensor is, the larger the grid cell size may be, and a wider aerial zone requires a bigger gas source. Gas concentration measurement was done with the quadcopter in the hover state at the center of each cell during the 10 s sampling time. Each measurement consisted of 50 readings, used to calculate the mean value for that measurement. The setup of the experiment is shown in Figure 8.

Data analysis of the gas dispersion and the point source was done manually by moving the quadcopter in a pattern using a remote control (RC), at a fixed altitude of 1 m from the ground. When the quadcopter reached the center of the cell, it remained in hover mode, and an interrupt control system was sent through the RC to collect data. Continuous flight patterns were completed in the order of the data acquisition sequence shown in Figure 6. This driving method has been used for the aim of manual correction during the field test and also to minimize errors; thus, a more accurate gas dispersion post-processing algorithm has been achieved.

The test for sensor positioning was done based on the CFD result, as presented in Figure 4. With the gas sensor mounted on the middle-bottom frame, unstable data reading and sometimes even zero value readings resulted. On the other hand, by having the gas sensor placed on the front of the quadcopter, the airflow generated by the propeller always passed the gas beyond the sensor. In hover mode, the quadcopter always adjusts its position to a stable state and produces equal air velocity on each of the rotors. Each quadcopter’s propeller rotation generates a vortex that draws the air from above and directs it downwards, to the CO sensors. When sensors were placed in the front of the quadcopter, the gas sensor responded well. The recorded data was analyzed for two flight patterns, as follows:

### 4.1. Point Source

**Figure 9:**
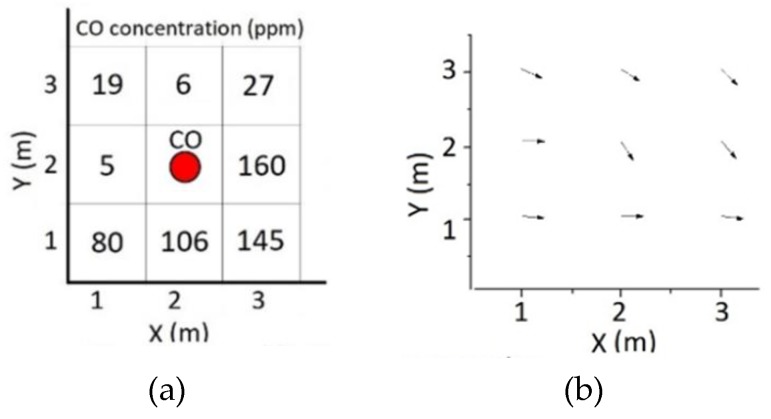

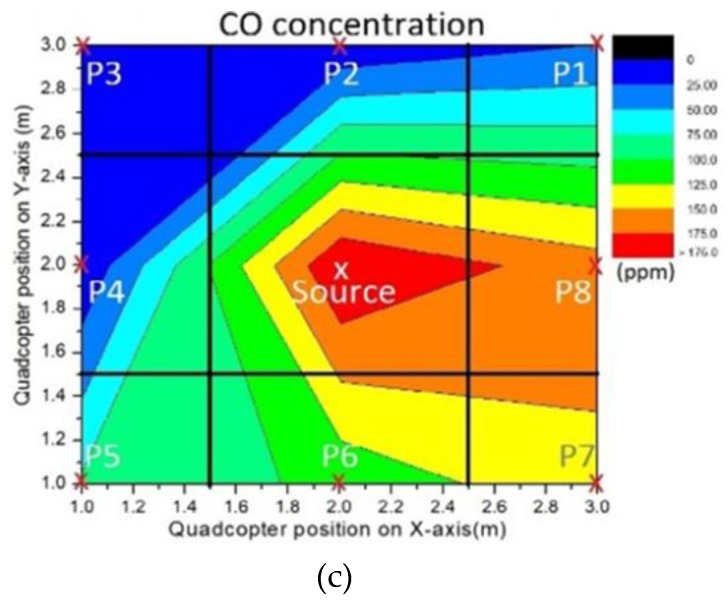
Point source experiment results: (**a**) CO concentrations; (**b**) CO vectors; (**c**) Gas dispersion.

In the first stage of the experiment, the sniffer quadcopter was used to determine the level of CO concentration in the surrounding atmosphere. The quadcopter flew in a square or circle pattern to read the CO concentration in the center of each of the eight sample points. As much as 8 sample points were measured during the test, and the total flight time of quadcopter was 130 s without landing and take-off time. Every time a position error occurred, an adjustment was made using Equations (4) and (8) under the condition that the point sample for one grid cell must be greater than one sample; thus, the variant and average value near each cell’s center can be obtained. Figure 9 shows the value of CO concentration measured for each cell. All data were distributed normally and the hypothesis from Equation (4) was applied to determine the vector of gas dispersion.

The analysis result for the gas dispersion vector with respect to the gas source position {(X,Y)=2} showed that the vector was {(X,Y)>2}. The carbon reading was modeled using a 2D contour in order to see the vector more clearly. The CO reading correlated with location was formulated in a matrix form shown in Equation (15).

(15) $$G_{i}^{k}=\left[\begin{array}{ccc} 19 & 6 & 27\\ 5 & \left(>160\right) & 160\\ 80 & 106 & 145 \end{array}\right]$$

The gas dispersion contour along the *x* and *y* axes showed that the dispersion started from the highest to the lowest concentration, more precisely from the cell’s center to the P1 point. In the point source method, the wind model of gas dispersion could not be seen clearly.

ANOVA analysis was used to compare cells two by two in all possible combinations, to get the estimation of CO concentration in the source. The analysis yielded the mean value (ΔG=6.89) , the standard deviation $\left(\sigma_{0}=0.36\right)\;$. After that, based on the gas dispersion vector, the irrelevant values were eliminated. To calculate the gas source concentration, the values from the first column (X=1) and the first row (Y=1) were eliminated. The sample points (P6, P7, P8) were used in the calculation since they were in accordance with the vector. The gas concentrations in each cell were summed up with the standard deviation value, and the results were averaged to get the CO source value, found to be 200.29 ppm. In comparison with real measurements of the gas source with sensors only, as much as 8.85% error was detected for CO concentration.

### 4.2. Non-Point Source

The non-point source experiment was done by collecting 24 sample points of CO concentration during 360 s of flight time. Data acquisition was done in the same manner used for the point source method. Two types of MOX sensors were used, i.e., MQ-135 and MQ-2 which were placed together on the quadcopter’s front frame. Both sensors were used simultaneously to ascertain the validity of the data and to get better analysis over the gas dispersion. In the field test, some burning coals were used as the CO source, placed on the position (X, Y) = (5, 2.5). Data of CO concentration were saved in the matrix form as presented in Equations (16) and (17). The CO concentration of the source was predicted, and the location of the source was analyzed through gas dispersion using the variant data of each cell.

(16) $$S_{MQ135}=\left[\begin{array}{cccccc} 19 & 19 & 19 & 35 & 82 & 40\\ 24 & 44 & 44 & 67 & 110 & 28\\ 49 & 18 & 16 & 18 & 33 & 28\\ 16 & 17 & 25 & 19 & 20 & 23 \end{array}\right]$$

(17) $$S_{MQ2}=\left[\begin{array}{cccccc} 37 & 30 & 27 & 39 & 65 & 45\\ 34 & 67 & 71 & 70 & 98 & 34\\ 54 & 33 & 29 & 42 & 38 & 18\\ 29 & 25 & 28 & 13 & 24 & 34 \end{array}\right]$$

**Figure 10:**
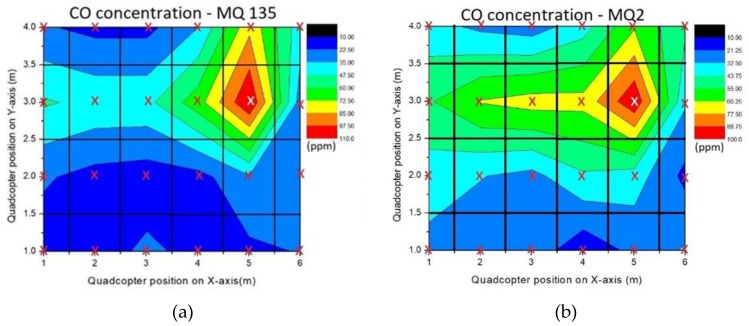
Non-point source experiment results in a 2D contour: (**a**) for MQ135 sensor; (**b**) for MQ2 sensor.

All 24 sample points in the matrix were computed, yielding a 2D gas concentration contour for each of the sensors, presented in Figure 10.

Based on the highest value of CO, the gas tended to drift toward the S13 cell or (X<5) in the horizontal direction and toward the S20 cell or (Y>3) in the vertical direction. Tentatively, it can be concluded that the gas source was around the red zone on the 2D contour. Further analysis using Equation (10) was done to discover the connection between gas dispersion and wind effect. A gentle breeze of 5 m s^−1^ blew around the field test area. Variant data of each sample was distributed normally to obtain standard deviation in form of kernel width for wind speed. Uncertainty of wind was conditioned as non-constant wind flow and represented by the (γ) parameter. Its value was estimated at 0.4 m based on the highest concentration stretch point and affected the stretch kernel shape. The calculation resulted in $\sigma_{o}\left(MQ135\right)=0.247$ and $\sigma_{o}\left(MQ2\right)=0.236$ with rotation vector $R_{1}\left(\alpha \right)=0$ and R2(α)=π2 , thus a wind vector with range value a = 2.24–2.25 m and b = 0.08 m was obtained. From the analysis of wind direction and gas dispersion, two possible positions for CO source resulted, which were at the coordinates (5≤x≤6.12), (2.92≤y≤3.08) and (4.92≤x≤5.0),(1.88≤y≤3) . Compared with the real position of the gas sensor (X,Y)=(5,2.5) , it can be concluded that only one out of the two possibilities is the source of pollutant area; bivariate Gaussian kernel analysis was used to assess the source location and minimize the error of CO reading.

## 5. Conclusions and Future Work

Air monitoring using a sniffer quadcopter with a flight pattern was designed to measure CO concentration in each cell. The post-processing analysis was used to determine the source location and its CO concentration. The results from CFD and the field test showed that the sensor placed in front of the frame (x>100) mm of the quadcopter was able to utilize the air trajectories generated by the propeller to direct the gas straight to the sensor. The best result was achieved when the data acquisition was made in hover mode to ensure constant airflow. Data acquisition was made using two methods, i.e., point source and non-point source. The point source method with a known location of the gas source was done using eight sample points forming a square pattern, having the source in the middle, which also is useful in facing the unpredictable wind effect.

The concentration of CO in the source was quantified using post-analysis by the means of the ANOVA method which was ran on eight samples during 130 s of flight time. Compared to the real data, the analysis showed as much as 8.85% error.

The other method was the non-point source, used to pinpoint the location of the gas source and also its concentration. This method adapts a grid pattern with 24 cells to collect data of CO with two types of gas sensors used simultaneously to ascertain data validity. The gas dispersion analysis results showed that the gas dispersion vector had changed twice, thus indicating two possible positions for gas source location. The gas dispersion vector has been analyzed using both the measurement position and CO concentration matrices. The readings of both sensors showed the same gas dispersion pattern, indicating the highest value of CO was in the S17 cell. The differences in the accuracy of data reading were affected only by the sensitivity of the MOX sensors. The correlation between gas dispersion and wind behavior must be known to overcome the possibility of result misinterpretation due to wind influence. Logically, the gas source must be in the vicinity of the cell having the highest CO concentration. A bivariate Gaussian kernel has been used to locate this cell’s position. The gas source was calculated with the same method used in the case of the point source; thus, the weight cell in the form of standard deviation was obtained with the value of CO between 118.06–133.24 ppm while the actual value was 125 ppm. Overall, the field tests were done by manipulating the gas source; the quadcopter’s altitude maintained at 1 m from the ground to collect data which then were calculated accordingly to acquire the gas source location. For the case of a small amount of burning coal, the experiment was possible only at low altitude with manual control of the quadcopter flight. The sensitivity of the sensors must pass the reliability test before being placed on the quadcopter because it affects the size of the cells. Finally, the analyses using normal dispersion and ANOVA were essential to obtain the gas concentration and gas source position.

This study using sniffer quadcopter has been limited to carbon monoxide measurements. The measurement method and gas source location detection method still have room for improvement. In the future, other pollutant compounds will be investigated and different gas sensors such as optical sensors will be used in comparison with the tested sensors. There are various application of mapping and measurement using a sniffer quadcopter, such as gas pipe leakage measurement, early warning systems for volcanic-prone areas, water pollution mapping with the pattern method, etc. From the perspective of flight pattern, with improvements in sensitivity and accuracy of reading, a larger scale grid pattern can be designed to save time on the data collection process. Quadcopter capability to withstand wind effects or heatwaves from the gas source can also be more developed. The higher goals are to utilize sniffer quadcopter as unmanned aerial security patrols to cope with environmental issues and monitor dangerous zones. In future experiments, we intend to test the data collection from the sensor at larger scales in quasi-real situations and also to use multiple drones which can communicate with each other and better map the field of interest.

## Figures and Tables

**Table 1:** Detail specifications of sniffer quadcopter.

Sniffer Quadcopter	Specifications
Frame material	Carbon fiber
Frame size	460 mm × 460 mm × 140 mm
	(width × length × height)
Propeller size	13 inches
Motor (rpm/V) Flight time	380 ± 40 min
Max. carry load	2460 gr
	(including quadcopter weight)
